# Discriminant content validity of a theoretical domains framework questionnaire for use in implementation research

**DOI:** 10.1186/1748-5908-9-11

**Published:** 2014-01-15

**Authors:** Johanna M Huijg, Winifred A Gebhardt, Mathilde R Crone, Elise Dusseldorp, Justin Presseau

**Affiliations:** 1 Clinical, Health, and Neuropsychology, Leiden University, Leiden, Wassenaarseweg 52, the Netherlands; 2 Department of Public Health and Primary Care, Leiden University Medical Center, Hippocratespad 21, Leiden, the Netherlands; 3 Netherlands Organization for Applied Scientific Research (TNO), Wassenaarseweg 56, Leiden, the Netherlands; 4 Institute of Health and Society, Newcastle University, Baddiley-Clark Building, Richardson Road, Newcastle upon Tyne, UK

**Keywords:** Implementation, Theoretical domains framework, Discriminant content validity, Questionnaire development

## Abstract

**Background:**

To improve the implementation of innovations in healthcare settings, it is important to understand factors influencing healthcare professionals’ behaviors. We aimed to develop a generic questionnaire in English and in Dutch assessing the 14 domains of behavioral determinants from the revised TDF (Cane *et al.*, 2012) that can be tailored to suit different targets, actions, contexts, and times of interest, and to investigate questionnaire items’ discriminant content validity.

**Methods:**

We identified existing questionnaires including items assessing constructs within TDF domains and developed new items where needed. Nineteen judges allocated 79 items to one or more TDF domains. One-sample t-tests were used to examine the discriminant content validity of each item, *i.e.*, whether items measured intended domains or whether items measured a combination of domains.

**Results:**

We identified items judged to discriminately measure 11 out of 14 domains. Items measuring the domains *Reinforcement*, *Goals*, and *Behavioral regulation* were judged to measure a combination of domains.

**Conclusions:**

We have developed a questionnaire in English and in Dutch able to discriminately assess the majority of TDF domains. The results partly support Cane *et al.*’s (2012) 14-domain validation of the TDF and suggest that Michie *et al.*’s (2005) 12-domain original version might be more applicable in developing a TDF-based questionnaire. The identified items provide a robust basis for developing a questionnaire to measure TDF-based determinants of healthcare professionals’ implementation behaviors to suit different targets, actions, contexts, and times. Future research should investigate the concurrent and predictive validity and reliability of such a questionnaire in practice.

## Background

Healthcare professionals routinely deliver pharmacological and behavior change interventions to their patients to promote health and prevent disease. However, as the evidence-base for effective interventions is continuously developing, the transfer of such evidence into routine practice often does not happen as desired [1-3]. For example, primary care-based interventions for increasing physical activity (PA) are effective [4-7], yet rates of PA counseling by healthcare professionals are suboptimal [8,9], as is the fidelity of delivery of PA interventions [2,10,11]. This gap between research and practice reduces the impact that effective behavior change interventions can have on public health [12,13]. Implementation research aims to bridge this gap by investigating methods to promote healthcare professionals’ uptake of research findings, including the study of factors influencing healthcare professional behavior [14,15].

Improving the adoption and implementation of evidence-based interventions into routine practice involves changes in healthcare professionals’ behaviors that may be influenced by a range of individual, organizational, and social factors [16-20]. Identifying the key factors associated with healthcare professional behavior can provide a basis for developing interventions to help healthcare professionals to use research findings more effectively [14]. Given the range of potential factors associated with behavior, many advocate the use of theory to guide the selection of factors to investigate [15,21-23]. In addition, the UK Medical Research Council guidance on developing and evaluating complex interventions recommends the use of theory in the intervention development phase [24]. The advantages of a theory-based approach are numerous: theory allows for a shared understanding, for the development of a cumulative science that limits the re-invention of existing concepts, and importantly is based on constructs which have been investigated, for which measures can be validated and standardized and have been shown to provide a useful account of behavior [25]. Furthermore, investigating the relationship between theory-based factors and healthcare professional behavior provides an opportunity to identify factors that can be targeted by implementation interventions to change healthcare professional behavior [15,23,26,27].

The number and heterogeneity of potential theories that might be used to guide implementation research poses a challenge to researchers wanting to assess and identify theory-based factors underlying healthcare professional behavior [22,28-30]. The Theoretical Domains Framework (TDF) [31] was developed as an integrative framework of theories of behavior change to overcome these challenges. The framework includes 12 theoretical domains of potential behavioral determinants and provides exemplar questions for the theoretical assessment of implementation problems. The framework has been used in a number of studies and was demonstrated to be useful for the development of qualitative [32,33] and quantitative [34-36] measurement tools to assess potential implementation behavior determinants. However, factor analysis implied that only one out of these three questionnaires was able to measure the theoretical domains independently [36]. Furthermore, the questionnaires were developed to assess determinants of specific implementation behaviors in specific settings (*i.e.*, tobacco use prevention and smoking cessation in dental healthcare [34], smoking cessation in maternal care [35], and different types of patient safety behaviors in hospitals [36]) and internal consistency reliability was low [34] or could be improved [35,36].

Since its original development, the consensus study that produced the TDF [31] has been validated, leading to Cane *et al.*’s [37] refined TDF. It extends the original TDF to include the following 14 domains: *Knowledge*; *Skills*; *Social/professional role and identity*; *Beliefs about capabilities*; *Optimism*; *Beliefs about consequences*; *Reinforcement*; *Intentions*; *Goals*; *Memory, attention and decision processes*; *Environmental context and resources*; *Social influences*; *Emotions*; and *Behavioral regulation*. Main differences between the original and the revised framework include the separation of the domain *Optimism* from the domain *Beliefs about capabilities* and the domain *Reinforcement* from the domain *Beliefs about consequences*. In addition, the domain *Motivation and goals* was divided into two separate domains, *i.e.*, *Intentions* and *Goals*, and the domain *Nature of the behaviors* was omitted in the revised framework. Although the framework is suggested to be useful for the development of theory-based questionnaires for use in implementation research, the content of the TDF has not yet been validated on item level. Therefore, it is not clear whether questionnaire items based on this recent version of the framework will be able to measure the 14 domains independently.

In the present study we aimed to develop a questionnaire assessing the 14 TDF domains, worded in such a way to provide researchers the capacity to tailor the items to the targets, actions, contexts and times of interest [38], whilst retaining the essential theoretical content in each item. Furthermore, we aimed to test the discriminant content validity of each item within the questionnaire.

## Methods

### Participants

Fifty-eight academics from the Netherlands were approached with details of the study and nineteen agreed to participate (response rate of 33%). They were either involved as experts in the field of behavior change, development of health behavior change interventions, or implementation of interventions in healthcare settings. They were recruited via the authors’ networks. The sample size was based on estimates of between three and 20 participants as adequate for judgment tasks [39,40]. We included academics (instead of healthcare professionals) in this study, because the discriminant content validation (DCV) exercise of allocating items to TDF domains requires theoretical knowledge and experience with the specific domains.

### Materials

We developed a questionnaire that initially included 79 items assessing each of the domains through their related key constructs (see Additional file 1). Constructs within domains were selected based on conceptual relatedness to the content of the domain (*i.e.*, Knowledge, Procedural knowledge, Skills, Professional role, and Memory); inclusion in relevant theories frequently used in the field of behavior change (and thus ready access to existing items): the Theory of Planned Behavior [41] (*i.e.*, Perceived behavioral control, Attitudes, Subjective norm, and Intention) and Social Cognitive Theory [42] (*i.e.*, Self-efficacy, Outcome expectancies, and Social support); existence of validated scales (*i.e.*, Optimism, Pessimism, Action planning, Attention, Affect, Stress, Automaticity, and Self-monitoring); and/or relevance to the implementation of PA interventions in routine healthcare by mapping factors resulting from previous research [43,44] onto the TDF domains. JP and JMH independently identified that the constructs Reinforcement, Priority, Resources/materials, and Descriptive norm were salient in the previous PA-based research and thus these constructs were also included as construct-indicators of their respective domains.

Items measuring constructs within the domains *Knowledge*, *Beliefs about capabilities*, *Optimism*, *Beliefs about consequences*, *Intentions*, *Social influences*, *Emotion*, and *Behavioral regulation* were adapted from previously published questionnaires (*i.e.*, [34,35,41,42,45-53]). Given lack of available questionnaires in the literature for some domains, new items were created for the domains *Skills*, *Social/professional role and identity*, *Reinforcement*, and *Environmental context and resources*. With regard to the domain *Goals*, items were newly developed for the construct Priority (as none could be located in the literature), while items measuring the construct Action planning were adapted from a previously published questionnaire [46]. With regard to the domain *Memory, attention, and decision making*, items measuring the construct Attention were adapted from a previously published questionnaire [51] and items measuring the construct Memory were newly developed. New items were developed based on discussions between JP and JMH. These discussions were informed by the academic literature on the concept and definition of specific domains and constructs, questions to identify behavior change processes as formulated by Michie *et al.*[31], and themes emerging from interviews on the implementation of PA interventions [43]. WAG and MRC supervised the development of the questionnaire and reviewed items’ face validity.

To develop a questionnaire which could be used by researchers in different fields of implementation research, items were formulated in a generic way using a '[action] in [context, time] with [target]’ construction based on the 'TACT principle’ [38], whereby researchers can specify the target, action, context, and time relevant to their research. The questionnaire was developed in English, then translated to Dutch and back-translated to English by an independent translator. The small amount of differences between the original and back-translated version of the questionnaire were discussed and adaptations were made.

### Procedure

In May and June 2012 participants were sent an email including the link to the online DCV exercise [54,55]. After one and two weeks non respondents received a reminder. Participants were provided with the aim of the study and an explanation of the DCV exercise. Then, they were asked to report their expertise on each of the 14 TDF domains on a 7-point Likert scale (1 = I am a layman with regard to this domain; 7 = I am an expert with regard to this domain).

We used Cane *et al.*’s [37] definitions of the 14 TDF domains (see Table 1), which were presented at the top of each rating page. The items of the questionnaire were listed below the definitions, in a random order. Participants were asked to consider carefully the meaning of each item and allocate it to the domain they perceived the item measures using the domain definitions provided. To determine whether items were deemed to discriminately measure domains or if they measure a combination of domains, participants were asked to allocate each of the 79 items to up to three domains. Upon allocating items, judges were asked to rate their confidence in each allocation between 0% and 100% (0% = not at all confident; 100% = extremely confident). For example, a judge could allocate an item to the domain *Knowledge* and rate their confidence 60% and allocate the same item to the domain *Skills* and rate their confidence 20%.

**Table 1:** **Definitions of the domains of the TDF**[37]^**1**^

**Domain**	**Definition**
D1 Knowledge	An awareness of the existence of something
D2 Skills	An ability or proficiency acquired through practice
D3 Social/professional role and identity	A coherent set of behaviors and displayed personal qualities of an individual in a social or work setting
D4 Beliefs about capabilities	Acceptance of the truth, reality, or validity about an ability, talent, or facility that a person can put to constructive us
D5 Optimism	The confidence that things will happen for the best or that desired goals will be attained
D6 Beliefs about consequences	Acceptance of the truth, reality, or validity about outcomes of a behavior in a given situation
D7 Reinforcement	Increasing the probability of a response by arranging a dependent relationship, or contingency, between the response and a given stimulus
D8 Intentions	A conscious decision to perform a behavior or a resolve to act in a certain way
D9 Goals	Mental representations of outcomes or end states that an individual wants to achieve
D10 Memory, attention and decision processes	The ability to retain information, focus selectively on aspects of the environment and choose between two or more alternatives
D11 Environmental context and resources	Any circumstance of a person’s situation or environment that discourages or encourages the development of skills and abilities, independence, social competence, and adaptive behavior
D12 Social influences	Those interpersonal processes that can cause individuals to change their thoughts, feelings, or behaviors
D13 Emotion	A complex reaction pattern, involving experiential, behavioral, and physiological elements, by which the individual attempts to deal with a personally significant matter or event
D14 Behavioral regulation	Anything aimed at managing or changing objectively observed or measured actions

^1^As described in Cane *et al.*[37] definitions are based on definitions from the American Psychological Associations’ Dictionary of Psychology [56].

### Data analysis

#### Classification of items

Ratings for matching items and domains (*i.e.*, items judged to assess the domain they were designed to assess) were coded 1 (a 'match’), whereas items judged to assess a different domain were coded -1 (a 'no match’); missing variables were scored 0. Each judgment was multiplied by its accompanied confidence rating (e.g., .20, .40, .80). As a consequence, the weighted judgments ranged from -1 to 1.

### DCV analysis

Following Dixon *et al.*[54,55], we used one-sample one-tailed *t*-tests to investigate whether each item was classified by the judges to represent the domain that the item aimed to measure. Judges were provided with three possibilities to allocate an item to a domain, therefore, the sum of the three weighted judgments was used for the one-sample *t*-tests. An item was classified as measuring a domain if its weighted judgment against that domain was significantly greater than zero (*p* < .05) [54]. The false discovery rate controlling procedure [57] was used to correct for multiple tests. Items that were classified to the correct (*i.e.*, intended) domain were included in the final questionnaire, whereas items that were allocated to more than one domain or that were classified to a domain other than the intended domain were not included. Analyses were performed in IBM SPSS Statistics version 19.0 [58].

### Inter-rater agreement

A generalization of Cohen’s kappa (*i.e.*, Light’s Kappa [59]) was calculated to assess agreement between judges across their allocation of all items to domains. For this calculation, we used the first domain that judges selected to represent the item. This was justified as the data indicated that judges used the first selected domain as the most preferable domain (*i.e.*, domain with the highest confidence ratings) to allocate an item to. As a consequence, the 79 items were scored between 1 and 14 (representing the domain it was allocated to) for each judge separately. This resulted in a data matrix composed of 79 rows (*i.e.*, the items) and 19 columns (*i.e.*, the judges). We also assessed inter-rater agreement for allocation of items to each domain. For this calculation, the 79 items were scored between 1 and 0 for each domain separately (representing if it was selected to the specific domain or not) and for each judge separately. This resulted in 14 data matrices, one for each domain, consisting of 79 rows and 19 columns. These analyses were repeated for the final set of items that was selected based on the DCV analysis. In line with previous research, κ-values of between .00 and .20 were labeled as slight agreement, values from .21 to .40 as fair agreement, values from .41 to .60 as moderate agreement, values from .61 to .80 as substantial, and values from .81 to 1.00 as almost perfect [60]. Analyses were performed in the R software environment [61], using the R-package 'Psy’ [62].

### Ethics

The Medical Ethics Committee of the Leiden University Medical Centre gave ethics approval for this study (reference number NV/CME 09/081).

## Results

### Judges’ expertise in the use of domains

Descriptive statistics of judges’ expertise in the use of each domain are shown in Table 2. Mean scores indicated that judges had at least some expertise on each domain. On average, judges rated that they had most expertise on the domains *Intentions* and *Goals*, whereas lowest expertise ratings were given to the domains *Social/professional role and identity*, and *Memory, attention, and decision processes*. Only three judges indicated to be a layman on, respectively, one, two, and seven domains.

**Table 2:** Judges’ expertise on domains

**Domains**	**Mean (SD)**
D1 Knowledge	4.63 (1.01)
D2 Skills	5.21 (0.71)
D3 Social/professional role and identity	3.47 (1.81)
D4 Beliefs about capabilities	5.26 (1.45)
D5 Optimism	3.68 (1.70)
D6 Beliefs about consequences	4.68 (1.49)
D7 Reinforcement	4.63 (1.50)
D8 Intentions	5.53 (1.31)
D9 Goals	5.47 (1.02)
D10 Memory, attention, and decision processes	3.58 (1.68)
D11 Environmental context and resources	4.11 (2.08)
D12 Social influences	5.32 (1.20)
D13 Emotion	4.11 (1.60)
D14 Behavioral regulation	5.26 (1.45)

*Note.* 1 = I am a layman with regard to this domain, 7 = I am an expert with regard to this domain.

Neither judges’ expertise with TDF domains nor their academic level (*i.e.*, PhD student, PhD, Professor) was related to their performance on the classification of items to domains calculated as the number of 'matches’. Pearson’s correlations were respectively *r* = -.35 (*p* = .14) and *r* = -.16 (*p* = .52).

## DCV results

Table 3 shows the results of the DCV analysis. Of 79 items, 32 were classified as measuring the intended domain and therefore included in the final questionnaire. Forty-seven items were allocated to more than one domain, of which 39 items were allocated to the intended domain as well as additional domains, while eight items were classified as measuring a domain other than the item aimed to measure. Table 4 shows Kappa values for the agreement between judges based on all 79 items of the initial questionnaire and the 32 items included in the final questionnaire. The final lists of items measuring TDF domains are shown in Table 5 (English) and Table 6 (Dutch).

**Table 3:** DCV analysis of the questionnaire

**Domain**	**Construct**	**Item**	**Mean**	***t*-value**	**Domain allocation if not classified to right domain (> 1)**
D1 Knowledge	Knowledge (3)	I am aware of the content and objectives of [innovation/guideline]	.82	9.99*	-
		I know the content and objectives of [innovation/guideline]	.88	17.58*	-
		I am familiar with the content and objectives of [innovation/guideline]	.82	8.76*	-
	Procedural knowledge (3)	I am aware of how to [A] in [C, T] with [Ta]	.74	5.51*	-
		I know how to [A] in [C, T] with [Ta]	.20	1.03	*D1*, D2, D4, D14
		I am familiar with how to [A] in [C, T] with [Ta]	.44	2.63	*D1*, D2
D2 Skills	Skills (4)	I have been trained how to [A] in [C, T] with [Ta]	.86	16.42*	-
		I have the proficiency to [A] in [C, T] with [Ta]	-.01	-0.05	*D2*, D4
		I have the skills to [A] in [C, T] with [Ta]	.53	3.84*	-
		I have practiced [A] in [C, T] with [Ta]	.67	5.28*	-
D3 Social/professional role and identity	Professional role (4)	[A] in [C, T] with [Ta] is part of my work as a [profession]	.85	9.62*	-
		As a [profession], it is my job to [A] in [C, T] with [Ta]	.89	29.74*	-
		It is my responsibility as a [profession] to [A] in [C, T] with [Ta]	.82	7.90*	-
		Doing [A] in [C, T] with [Ta] is consistent with my [profession]	.81	8.70*	-
D4 Beliefs about capabilities	Self-efficacy (2)	I am confident that I can [A] in [C, T] with [Ta] even when [Ta] is not motivated	71	5.80*	-
		I am confident that I can [A] in [C, T] with [Ta] even when there is little time	.67	5.31*	-
	Perceived behavioral control (4)	I am confident that if I wanted I could [A] in [C, T] with [Ta]	.78	7.07*	-
		How much control do you have over [A] in [C, T] with [Ta]?	.02	0.11	*D4*, D2, D14
		For me, [A] in [C, T] with [Ta] is… (Very difficult – very easy)	.40	2.17	*D4*, D2, D5
		For me, [A] in [C, T] with [Ta] is… (Impossible – possible)	.33	2.21	*D4*, D2, D5, D6
D5 Optimism	Optimism (3)	With regard to [A] in [C, T] with [Ta] in uncertain times, I usually expect the best	.64	5.01*	-
		With regard to [A] in [C, T] with [Ta] I’m always optimistic about the future	.65	4.35*	-
		With regard to [A] in [C, T] with [Ta] overall, I expect more good things to happen than bad	.13	0.65	*D5*, D6
	Pessimism (3)	With regard to [A] in [C, T] with [Ta] if something can go wrong, it will	.43	3.13	*D5*, D4, D6
		With regard to [A] in [C, T] with [Ta] I hardly ever expect things to go my way	.03	0.14	*D5*, D4, D6
		With regard to [A] in [C, T] with [Ta] I rarely count on good things happening to me	.44	2.60	*D5*, D6
D6 Beliefs about consequences	Attitudes (2)	For me, [A] in [C, T] with [Ta] is… (Useless – useful)	.45	2.71	*D6*, D5
		For me, [A] in [C, T] with [Ta] is… (bad – good)	.42	2.82	*D6*, D1, D3
	Outcome expectancies (2)	If I [A] in [C, T] with [Ta] it will benefit public health	.60	4.44*	-
		If I [A] in [C, T] with [Ta] it will have disadvantages for my relationship with [Ta]	.58	4.14*	-
D7 Reinforcement	Reinforcement (3)	Whenever I [A] in [C, T] with [Ta], I get financial reimbursement	.42	2.38	*D7*, D6
		Whenever I [A] in [C, T] with [Ta], I get recognition from professionals who are important to me	-.51	-3.77*	*D7*, D3, D6, **D12**
		Whenever I [A] in [C, T] with [Ta, I feel like I am making a difference	-.68	-7.14*	*D7*, D4, **D6**, D13
D8 Intentions	Intention (4)	For how many of the next 10 [Ta] do you intend to [A] in [C]?	.73	6.92*	-
		I will definitely [A] in [C] with [Ta] in the next [T]	.63	3.89*	-
		I intend to [A] in [C] with [Ta] in the next [T]	.66	5.83*	-
		How strong is your intention to [A] with [Ta] in [C] in the next [T]?	.89	20.60*	-
D9 Goals	Action planning (4)	I have a clear plan of how I will [A] in [C, T] with [Ta]	.47	2.75	*D14*, D8, *D9*
		I have a clear plan under what circumstances I will [A] in [C, T] with [Ta]	.26	1.22	*D14*, D8, *D9*
		I have a clear plan when I will [A] in [C, T] with [Ta]	.43	2.26	*D14*, D8, *D9*
		I have a clear plan how often I will [A] in [C, T] with [Ta]	-.18	-0.83	*D14*, D8, *D9*
	Priority (4)	Generally, in [C, T] with [Ta], how often is covering something else on your agenda a higher priority than [A]	-.58	-4.10*	*D9*, D3, **D10**, D11
		Generally, in [C, T] with [Ta], how often does covering something else on your agenda take precedence over [A]	-.58	-4.30*	*D9*, D3, **D10**, D11
		Generally, in [C, T] with [Ta], how often is covering something else on your agenda more urgent than [A]	-.49	-4.32*	*D9*, D3, **D10**, **D11**, D14
		Generally, in [C, T] with [Ta], how often is covering something else on your agenda more pressing than [A]	-.63	-4.82*	*D9*, D3, **D10**, D11
D10 Memory, attention and decision processes	Memory (4)	[A] in [C, T] with [Ta] is easy to remember	.32	1.64	*D10*, D1, D4
		How often do you forget [A] in [C, T] with [Ta]?	.63	4.55*	-
		How often do you have to check the [innovation/guideline] before [A] in [C, T] with [Ta]?	-.66	-5.52*	*D10*, D1, **D4**
		To what extent do you know [innovation/guideline] by heart to [A] in [C, T] with [Ta]?	-.91	-30.09*	**D1**
	Attention (4)	When I need to concentrate to [A] in [C, T] with [Ta], I have no trouble focusing my attention	.77	7.10*	-
		When I am working hard on [A] in [C, T] with [Ta], I still get distracted by events around me	.52	3.24	-
		When trying to focus my attention on [A] in [C, T] with [Ta], I have difficulty blocking out distracting thoughts	.68	5.06*	-
		When concentrating on [A] in [C, T] with [Ta], I can focus my attention so that I become unaware of what’s going on around me	.68	6.03*	-
D11 Environmental context and resources	Resources/material (8)	[Innovation/guideline] has a good fit with routine practice	.22	1.44	*D11*, D1, D3
		[Innovation/guideline] provides the possibility to adapt it to the [Ta]’s needs (*e.g.*, culture)	.25	1.55	*D11*, D3, D6, D12
		In the organization I work [A] in [C, T] with [Ta] is routine	-.02	-0.11	*D11*, D2, D3, D12, D14
		In the organization I work there is enough time to [A ] in [C, T] with [Ta]	.42	2.24	*D11*, D3
		Within the socio-political context there is sufficient financial support (*e.g.*, from local authorities, insurance companies, the government) for [innovation/guideline]	.86	13.48*	-
		Within the socio-political context there are good networks between parties involved in [innovation/guideline]	.74	9.35*	-
		Prior to delivery of [innovation/guideline] professionals are provided with a training to [A] in [C, T] with [Ta]	-.51	-3.29	*D11*, D2
		During the delivery of [innovation/guideline] professionals are provided with sufficient financial reimbursement to [A] in [C, T] with [Ta]	.13	0.69	*D11*, D7
D12 Social influences	Social support (4)	I can rely on the team of professionals with whom I deliver [innovation] when things get tough on [A] in [C, T] with [Ta]	-.35	-1.96	*D12*, D3, D11
		My colleagues are willing to listen to my problems related to [A] in [C, T] with [Ta]	.22	1.30	*D12*, D3, D11
		The team of professionals with whom I deliver [innovation] is helpful in getting [A] in [C, T] with [Ta] done	.14	0.74	*D12,* D11
		I can rely on my colleagues when things get tough on [A] in [C, T] with [Ta]	.07	0.38	*D12,* D3, D11
	Subjective norm (2)	Most people who are important to me think that I should [A] in [C, T] with [Ta]	.84	9.04*	-
		Most people whose opinion I value would approve me of [A] in [C, T] with [Ta]	.61	3.97*	-
	Descriptive norm (2)	The team of professionals with whom I deliver [innovation/guideline] [A] in [C, T] with [Ta]	.35	2.13	*D12,* D3, D11
		Respected colleagues [A] in [C, T] with [Ta]	.24	1.26	*D12,* D3, D11
D13 Emotion	Affect (2)	Thinking about yourself and how you normally feel as a professional that delivers [innovation/guideline], to what extent do you generally feel inspired with regard to [A] in [C, T] with [Ta]	-.09	-0.49	*D13*, D3, D14
		Thinking about yourself and how you normally feel as a professional that delivers [innovation/guideline], to what extent do you generally feel nervous with regard to [A] in [C, T] with [Ta]	-.01	-0.05	*D13*, D3, D4
	Stress (2)	Have you recently, during the past two weeks been able to enjoy your normal day-to-day activities?	.55	3.55*	-
		Have you recently, during the past two weeks been feeling unhappy and depressed?	.78	6.95*	-
D14 Behavioral regulation	Automaticity (2)	[A] in [C, T] with [Ta] is something I do automatically	-.31	-0.20	*D14*, D2, D10
		[A] in [C, T] with [Ta] is something I do without thinking	-.45	-3.29	D2, D10
	Self-monitoring (4)	I keep track of my overall progress towards [A] in [C, T] with [Ta]	.27	1.57	*D14*, D7, D9, D13
		I tend to notice my successes while working towards [A] in [C, T] with [Ta]	-.39	-2.53	*D14*, D9
		I am aware of my day-to-day behavior as I work towards [A] in [C, T] with [Ta]	-.09	-0.49	*D14*, D8, D9, D10
		I check regularly whether I am getting closer to attaining [A] in [C, T] with [Ta]	.49	3.45	*D14*, D9
	Action planning (4)	I have a clear plan of how I will [A] in [C, T] with [Ta]	.47	2.75	*D14*, D8, *D9*
		I have a clear plan under what circumstances I will [A] in [C, T] with [Ta]	.26	1.22	*D14*, D8, *D9*
		I have a clear plan when I will [A] in [C, T] with [Ta]	.43	2.26	*D14*, D8, *D9*
		I have a clear plan how often I will [A] in [C, T] with [Ta]	-.18	-0.83	*D14*, D8, *D9*

*Note.* [A], action; [C], context; [T], time; [Ta], target; *, significant at .05 level, after false discovery rate controlling procedure for multiple tests; D1, Knowledge; D2, Skills; D3, Social/professional role and identity; D4, Beliefs about capabilities; D5, Optimism; D6, Beliefs about consequences; D7, Reinforcement; D8, Intentions; D9, Goals; D10, Memory, attention, and decision processes; D11, Environmental context and resources; D12, Social influences; D13, Emotion; D14, Behavioral regulation; *D*, domain the item intended to measure; **D**, domain the item is systematically allocated to other than the item intended to measure.

**Table 4:** Light’s κ-values for all items and the items included in the final questionnaire

**Domains**	**All 79 items κ (95% C.I.)**	**32 final items κ (95% C.I.)**
All items and domains	.56 (.50–.62)	.82 (.79–.85)
D1 Knowledge	.76 (.63–.87)	.88 (.77–.96)
D2 Skills	.58 (.35–.71)	.80 (.73–.87)
D3 Social/professional role and identity	.59 (.37–.75)	.86 (.72–.93)
D4 Beliefs about capabilities	.55 (.41–.71)	.73 (.60–.81)
D5 Optimism	.60 (.49–.69)	.68 (.63–.72)
D6 Beliefs about consequences	.49 (.34–.62)	.70 (.67–.73)
D7 Reinforcement	.59 (.50–.68)	-
D8 Intentions	.75 (.56–.87)	.93 (.89-1.00)
D9 Goals	.11 (.07–.14)	-
D10 Memory, attention, and decision processes	.63 (.48–.75)	.85 (.79–.90)
D11 Environmental context and resources	.48 (.34–.65)	.82 (.73–.87)
D12 Social influences	.53 (.43–.67)	.78 (.69–.86)
D13 Emotion	.58 (.44–.70)	.90 (.83–.96)
D14 Behavioral regulation	.36 (.20–.52)	-

*Note.* C.I., biased-corrected bootstrapped confidence interval of Light’s Kappa (based on 200 bootstrap samples).

With regard to the 32 final items, κ-values could not be calculated for the domains *Reinforcement*, *Goals*, and *Behavioral regulation*, because none of the items measuring these domains was included in the final questionnaire.

**Table 5:** Final list of items measuring TDF domains (English)

**Domain**	**Item**
D1 Knowledge (4)	I am aware of the content and objectives of [innovation/guideline]
	I know the content and objectives of [innovation/guideline]
	I am familiar with the content and objectives of [innovation/guideline]
	I am aware of how to [A] in [C, T] with [Ta]
D2 Skills (3)	I have been trained how to [A] in [C, T] with [Ta]
	I have the skills to [A] in [C, T] with [Ta]
	I have practiced [A] in [C, T] with [Ta]
D3 Social/professional role and identity (4)	[A] in [C, T] with [Ta] is part of my work as a [profession]
	As a [profession], it is my job to [A] in [C, T] with [Ta]
	It is my responsibility as a [profession] to [A] in [C, T] with [Ta]
	Doing [A] in [C, T] with [Ta] is consistent with my [profession]
D4 Beliefs about capabilities (3)	I am confident that I can [A] in [C, T] with [Ta] even when [Ta] is not motivated
	I am confident that I can [A] in [C, T] with [Ta] even when there is little time
	I am confident that if I wanted I could [A] in [C, T] with [Ta]
D5 Optimism (2)	With regard to [A] in [C, T] with [Ta] in uncertain times, I usually expect the best
	With regard to [A] in [C, time] with [Ta] I’m always optimistic about the future
D6 Beliefs about consequences (2)	If I [A] in [C, T] with [Ta] it will benefit public health
	If I [A] in [C, T] with [Ta] it will have disadvantages for my relationship with [Ta]
D7 Reinforcement (0)	**
D8 Intentions (4)	For how many of the next 10 [Ta] do you intend to [A] in [C]?
	I will definitely [A] in [C] with [Ta] in the next [T]
	I intend to [A] in [C] with [Ta] in the next [T]
	How strong is your intention to [A] with [Ta] in [C] in the next [T]?
D9 Goals (0)	**
D10 Memory, attention and decision processes (4)	How often do you forget [A] in [C, T] with [Ta]?
	When I need to concentrate to [A] in [C, T] with [Ta], I have no trouble focusing my attention
	When trying to focus my attention on [A] in [C, T] with [Ta], I have difficulty blocking out distracting thoughts
	When concentrating on [A] in [C, T] with [Ta], I can focus my attention so that I become unaware of what’s going on around me
D11 Environmental context and resources (2)	Within the socio-political context there is sufficient financial support (*e.g.*, from local authorities, insurance companies, the government) for [innovation/guideline]
	Within the socio-political context there are good networks between parties involved in [innovation/guideline]
D12 Social influences (2)	Most people who are important to me think that I should [A] in [C, T] with [Ta]
	Most people whose opinion I value would approve me of [A] in [C, T] with [Ta]
D13 Emotion (2)	Have you recently, during the past two weeks been able to enjoy your normal day-to-day activities?
	Have you recently, during the past two weeks been feeling unhappy and depressed?
D14 Behavioral regulation (0)	**

*Note.* [A], action; [C], context; [T], time; [Ta], target; **, discriminant content validity of the items measuring these domains was not demonstrated.

**Table 6:** Final list of items measuring TDF domains (Dutch)

**Domain**	**Item**
D1 Knowledge (4)	Ik ben op de hoogte van de inhoud en doelstellingen van [innovatie/richtlijn]
	Ik ken de inhoud en doelstellingen van [innovatie/richtlijn]
	Ik ben bekend met de inhoud en doelstellingen van [innovatie/richtlijn]
	Ik ben op de hoogte van hoe ik [A] in [C, T] met [Ta]
D2 Skills (3)	Ik ben getraind hoe ik [A] in [C, T] met [Ta]
	Ik heb de vaardigheden om [A] in [C, T] met [Ta]
	Ik heb [A in [C, T] met [Ta] in [C, T] met [Ta] geoefend
D3 Social/professional role and identity (4)	[A] in [C, T] met [Ta] hoort bij mijn werk als [beroep]
	Als [beroep] is het mijn taak om [A] in [C, T] met [Ta]
	Het is mijn verantwoordelijkheid als [beroep] om [A] in [C, T] met [Ta]
	Het doen van [A] in [C, T] met [Ta] is overeenkomend met mijn [beroep]
D4 Beliefs about capabilities (3)	Ik heb er vertrouwen in dat ik in staat ben om [A] in [C, T] met [Ta], zelfs wanneer [Ta] niet gemotiveerd is
	Ik heb er vertrouwen in dat ik in staat ben om [A] in [C, T] met [Ta], zelfs wanneer er weinig tijd is
	Ik heb er vertrouwen in dat als ik het wil, ik in staat ben om [A] in [C, T] met [Ta]
D5 Optimism (2)	Als het gaat om [A] in [C, T] met [Ta] dan verwacht ik in onzekere tijden, toch meestal het beste
	Als het gaat om [A] in [C, T] met [Ta] dan ben ik altijd optimistisch over de toekomst
D6 Beliefs about consequences (2)	Als ik [A] in [C, T] met [Ta], dan zal dit voordelig zijn voor de publieke gezondheid
	Als ik [A] in [C, T] met [Ta], dan zal het nadelig zijn voor mijn relatie met [Ta]
D7 Reinforcement (0)	**
D8 Intentions (4)	Voor hoeveel van de komende 10 [Ta] heb je de intentie om [A] in [C]?
	Ik zal zeker [A] in [C] met [Ta] in de komende [T]
	Ik ben van plan om [A] in [C] met [Ta] in de komende [T]
	Hoe sterk is uw intentie om [A] in [C] met [Ta] in de komende [T]?
D9 Goals (0)	**
D10 Memory, attention and decision processes (4)	Hoe vaak vergeet u [A] in [C, T] met [Ta]?
	Als ik me moet concentreren om [A] in [C, T] met [Ta], lukt het mij gemakkelijk om mijn aandacht hierop te richten.
	Als ik mijn aandacht probeer te richten op [A] in [C, T] met [Ta], vind ik het moeilijk afleidende gedachten uit te schakelen
	Als ik me concentreer op [A] in [C, T] met [Ta], kan ik mijn aandacht zo richten dat ik niet merk wat er om me heen gebeurt
D11 Environmental context and resources (2)	Binnen de sociaal-politieke context is er voldoende financiële ondersteuning (bijv. van gemeente, zorgverzekeraars, de overheid) voor [innovatie/richtlijn]
	Binnen de sociaal politieke context zijn er goede netwerken tussen partijen betrokken bij [innovatie/richtlijn]
D12 Social influences (2)	De meeste mensen die belangrijk voor mij zijn vinden dat ik [A] in [C, T] met [Ta] zou moeten doen
	De meeste mensen van wie ik hun mening waardeer, zouden [A] in [C, T] met [Ta] goedkeuren
D13 Emotion (2)	Heeft u de laatste tijd (de afgelopen twee weken) plezier kunnen beleven aan gewone, dagelijkse bezigheden?
	Heeft u zich de laatste tijd (de afgelopen twee weken) ongelukkig en neerslachtig gevoeld?
D14 Behavioral regulation (0)	**

*Note.* [A], actie; [C], context; [T], tijd; [Ta], target; **, discriminant content validity of the items measuring these domains could was not demonstrated.

### Knowledge

The domain *Knowledge* was defined as 'an awareness of the existence of something’ [37]. Of the six *Knowledge* items included in the DCV exercise, four items were classified as measuring the domain *Knowledge* (Table 4) and were included in the final questionnaire. Two items were allocated to more than one domain. In addition to the domain *Knowledge*, these items were amongst others allocated to the domain *Skills*. The extent to which judges agreed on which items measured the domain was substantial when including all items (κ = .76; 95% C.I. .63-.87; Table 4) and almost perfect for the 32 final items (κ = .88; 95% C.I. .77-.96; Table 4).

### Skills

The domain *Skills* was defined as 'an ability or proficiency acquired through practice’ [37]. Three out of four *Skills* items included in the DCV were classified as measuring the intended domain (Table 3) and were included in the final questionnaire. In addition to the domain *Skills*, nine judges allocated the item 'I have the proficiency to…’ to the domain *Beliefs about capabilities*. With all items included, moderate agreement between judges was found for their allocation of items to the domain (κ = .58; 95% C.I. .35-.71; Table 4), while substantial agreement was found for the 32 final items (κ = .80; 95% C.I. .73-.87; Table 4).

### Social/professional role and identity

The domain *Social/professional role and identity* was defined as 'a coherent set of behaviors and displayed personal qualities of an individual in a social or work setting’ [37]. All four *Social/professional role and identity* items included in the DCV were classified as measuring the intended domain (Table 3) and were included in the final questionnaire. The extent to which judges agreed on which items measured the domain was moderate with all items included (κ = .59; 95% C.I. .37-.75; Table 4) and almost perfect for the 32 final items (κ = .86; 95% C.I. .72-.93; Table 4).

### Beliefs about capabilities

The domain *Beliefs about capabilities* was defined as 'acceptance of the truth, reality, or validity about an ability, talent, or facility that a person can put to constructive use’ [37]. Six *Beliefs about capabilities* items were included in the DCV exercise. The three items containing the word 'confident’ were classified as measuring the intended domain (Table 3) and were included in the final questionnaire. The items measuring the difficulty and possibility of [action] in [context, time] with [target] were allocated to more than one domain. In addition to the domain *Beliefs about capabilities*, they were often allocated to the domain *Skills*. The item 'How much control do you have over…’ was allocated to the intended domain, but also to the domains *Skills* and *Behavioral regulation*. With all items included, moderate agreement between judges was found for their allocation of items to the domain (κ = .55; 95% C.I. .41-.71; Table 4), while substantial agreement was found for the 32 final items (κ = .73; 95% C.I. .60-.81; Table 4).

### Optimism

The domain Optimism was defined as 'the confidence that things will happen for the best or that desired goals will be attained’ [37]. Two out of six *Optimism* items included in the DCV were classified as measuring the domain *Optimism* (Table 3). These were included in the final questionnaire. Four items were allocated to more than one domain, including the domains *Beliefs about capabilities* and *Beliefs about consequences*. The extent to which judges agreed on which items measured the domain was moderate with all items included (κ = .60; 95% C.I. .49-.69; Table 4) and substantial for the final 32 items (κ = .68; 95% C.I. .63-.72; Table 4).

### Beliefs about consequences

The domain *Beliefs about consequences* was defined as 'acceptance of the truth, reality, or validity about outcomes of a behavior in a given situation’ [37]. Of the four *Beliefs about consequences* items included in the DCV, only two items were classified as measuring the intended domain (Table 3) and included in the questionnaire. These were the items measuring the construct Outcome expectancies. The two items measuring the construct Attitudes were allocated to a variety of domains, including *Social/professional role and identity* and *Optimism*. With all items included, moderate agreement between judges was found for their allocation of items to the domain (κ = .49; 95% C.I. .34-.62; Table 4), while substantial agreement was found for the final 32 items (κ = .70; 95% C.I. .67-.73; Table 4).

### Reinforcement

The domain *Reinforcement* was defined as 'increasing the probability of a response by arranging a dependent relationship, or contingency, between the response and a given stimulus’ [37]. The DCV exercise included three items intended to measure *Reinforcement*, but none of them was classified as measuring the domain (Table 3) and so none of them was included in the final questionnaire. The item '…I get financial reimbursement’ was, in addition to the intended domain, allocated to the domain *Beliefs about consequences*. Two items were classified as measuring domains they were not intended to measure. The item '…I get recognition from professionals who are important to me’ was classified as measuring the domain *Social influences* and the item '…I feel like I am making a difference’ was classified as measuring the domain *Beliefs about consequences*. Five judges did not allocate any item to the domain. Without these judges taken into account Cohen’s kappa indicated moderate agreement (κ = .59; 95% C.I. .50-.68; Table 4).

### Intentions

The domain *Intentions* was defined as 'a conscious decision to perform a behavior or a resolve to act in a certain way’ [37]. All four items included in the DCV to measure *Intentions* were classified as measuring the domain (Table 3) and included in the final questionnaire. The extent to which judges agreed on which items measured the domain was substantial with all items included (κ = .75; 95% C.I. .56-.87; Table 4) and almost perfect for the final 32 items (κ = .93; 95% C.I. .89-1.00; Table 4).

### Goals

The domain *Goals* was defined as 'mental representations of outcomes or end states that an individual wants to achieve’ [37]. Eight *Goals* items were included in the DCV exercise. None of them were classified to the right domain (Table 3) and thus *Goals* items were not included in the final questionnaire. Items measuring the construct *Priority* were classified as measuring the domain *Memory, attention, and decision processes*. The four items measuring the construct Action planning were included in the DCV as measuring both the domain *Goals* and *Behavioral regulation*. They were not classified as measuring these two domains, because they were also often allocated to the domain *Intentions*. Three judges did not allocate items to the domain. Without these judges taken into account kappa indicated slight agreement (κ = .11; 95% C.I. .07-.14; Table 4).

### Memory, attention, and decision processes

The domain *Memory, attention, and decision processes* was defined as 'the ability to retain information, focus selectively on aspects of the environment and choose between two or more alternatives’ [37]. Eight items were included in the DCV exercise to measure the domain *Memory, attention, and decision processes*. Four of these items were classified to measure the intended domain (Table 3) and were included in the final questionnaire. Two items were allocated to more than one domain and two items measuring the construct Memory were classified as measuring a domain other than they were intended to measure (*i.e.*, *Knowledge* and *Beliefs about capabilities*). The extent to which judges agreed on which items measured the domain was substantial with all items included (κ = .63; 95% C.I. .48-.75; Table 4) and almost perfect for the final 32 items (κ = .85; 95% C.I. .79-.90; Table 4).

### Environmental context and resources

The domain *Environmental context and resources* was defined as 'any circumstance of a person’s situation or environment that discourages or encourages the development of skills and abilities, independence, social competence, and adaptive behavior’ [37]. Eight items were included in the DCV to measure this domain, while only two items were classified as measuring the domain (Table 3) and therefore could be included in the final questionnaire. Other items, not including the word 'socio-political context’ were, in addition to the intended domain, foremost allocated to the domains *Skills*, *Social/professional role and identity*, and *Social influences*. With all items included, moderate agreement between judges was found for their allocation of items to the domain (κ = .48; 95% C.I. .34-.65; Table 4), while almost perfect agreement was found for the final 32 items (κ = .82; 95% C.I. .73-.87; Table 4).

### Social influences

The domain *Social influences* was defined as 'those interpersonal processes that can cause individuals to change their thoughts, feelings, or behaviors’ [37]. Two out of eight *Social influences* items included in the DCV, were classified as measuring the intended domain (Table 3) and therefore included in the final questionnaire. These were the items measuring the construct Subjective norm. In addition to the domain *Social influences*, the other six items were mostly allocated to the domains *Social/professional role* and identity and *Environmental context and resources*. The extent to which judges agreed on which items measured the domain was moderate with all items included (κ = .53; 95% C.I. .43-.67; Table 4) and substantial for the final 32 items (κ = .78; 95% C.I. .69-.86; Table 4).

### Emotion

The domain *Emotion* was defined as 'a complex reaction pattern, involving experiential, behavioral, and physiological elements, by which the individual attempts to deal with a personally significant matter or event’ [37]. Of the four *Emotion* items included in the DCV exercise, the two items measuring the construct Stress were classified as measuring the intended domain (Table 3). These items were included in the final questionnaire. The two items measuring the construct Affect were allocated to more than one domain, including *Emotion*, *Social/professional role and identity*, and *Beliefs about capabilities*. With all items included, moderate agreement between judges was found for their allocation of items to the domain (κ = .58; 95% C.I. .44-.70; Table 4), while almost perfect agreement was found for the final 32 items (κ = .90; 95% C.I., .83-.96; Table 4).

### Behavioral regulation

The domain *Behavioral regulation* was defined as 'anything aimed at managing or changing objectively observed or measured actions’ [37]. Ten items, including Action planning items also aimed to measure the domain *Goals*, were included in the DCV to measure *Behavioral regulation*. None of them were classified to the right domain (Table 3) and therefore *Behavioral regulation* items were not included in the final questionnaire. The six items measuring the constructs Automaticity and Self-monitoring were allocated to more than one domain including *Behavioral regulation*, *Skills*, *Goals*, and *Memory attention, and decision processes*. Two judges did not allocate any of the 79 items to the domain. Without these judges taken into account kappa indicated fair agreement (κ = .36; 95% C.I. .20-.52; Table 4).

### All items and domains

Overall, moderate agreement was found for the allocation of all 79 items to the 14 domains (κ = .56; 95% C.I. .50-.62; Table 4), while almost perfect agreement was found for the allocation of the final 32 items to the 14 domains (κ = .82; 95% C.I. .79-.85; Table 4).

## Discussion

We have developed a TDF-based questionnaire in both English and Dutch able to discriminately assess the majority of domains. For the first time, items have been operationalized to assess TDF domains using theoretical constructs within each domain *and* these items were judged to be either pure measures of the domain, or else also measuring other domains. Our findings provide an additional level of validation for the content of the TDF: not only do judges agree about the constructs within each domain and the domain structure as demonstrated by Cane *et al.*[37], but the majority of TDF domains have now been shown to be largely discriminately measurable. These results correspond with Taylor *et al.*[36,63] who found good discriminant validity of TDF domains in a questionnaire measuring influences on patient safety behaviors [36] and in the Determinants of Physical Activity Questionnaire [63]. While Taylor *et al.*[36,63] used specific items (*i.e.*, related to a specific application), our items are generic and allow for application within a range of different contexts in which implementation research takes place. In summary, the development of our questionnaire provides important evidence of content validity and is a first step towards the development of a valid and reliable questionnaire to measure TDF-based factors underlying healthcare professionals’ specific implementation behaviors.

Of the 79 items assessed, 32 items were able to discriminately measure the following 11 domains: *Knowledge*, *Skills*, *Social/professional role and identity*, *Beliefs about capabilities*, *Optimism*, *Beliefs about consequences*, *Intentions*, *Memory, attention and decision processes*, *Environmental context and resources*, *Social influences,* and *Emotion*. For each of these domains at least two items were identified that can be used in the development of a TDF-based questionnaire.

Following judges allocations, items were not able to measure the domains *Reinforcement*, *Goals*, and *Behavioral regulation*. Items intended to measure these domains were allocated to multiple domains or classified to a domain other than the item intended to measure. This may be due to a few reasons. First, it is possible that the items used to operationalize the constructs within these domains were not appropriate, which might be related to the fact that some of *Reinforcement* and *Goals* items were newly developed by the researchers rather than previously-validated items. Nevertheless, items intended to measure the domain *Behavioral regulation* through the constructs Automaticity, Self-monitoring, and Action planning were adapted from previously published questionnaires, and thus it is unlikely that the existing level of validation of items is responsible for challenges in allocating items to particular domains. Second, it might be that items could not be classified to measure these three domains, because the domain definitions were not fit for purpose. This is associated with the finding that five, three, and two judges did not allocate any of the items to, respectively, the domains *Reinforcement*, *Goals*, and *Behavioral regulation*. The findings may also be explained by the use of domain definitions instead of construct definitions to allocate items, while items were previously developed to target individual constructs rather than broader domains. The allocation of items to domain definitions might therefore be influenced by the closeness of the definition of the domain to the definition of its constituent constructs. Finally, it could be that the remaining domains themselves cannot be discriminately measured. This seems a plausible explanation, as the domain *Reinforcement* is a refinement of the *Beliefs about consequences* domain and was originally included within the latter domain in the original TDF [31]. It is then perhaps not surprising that the *Reinforcement* items were judged to be assessing *Beliefs about consequences*, and arguably, such assignment is theoretically appropriate. Furthermore, the refinement of the domain *Motivation and goals* of the original TDF [31] into the domains *Goals* and *Intentions* in the recent version of the TDF and the classification of multiple goal-related constructs to the domains *Goals*, *Intentions*, and *Behavioral regulation* imply overlap between these domains. Therefore, it is perhaps also not surprising that the items measuring these domains were allocated to all three domains, and thus are not able to discriminately measure them. From a discriminant content validity perspective, taken together these results support keeping to the 12 original domains as a basis for the development of TDF questionnaires. When using the 12-domain framework [31] to develop a TDF-based questionnaire, items measuring the domains *Behavioral regulation* and *Nature of the behaviors* should be identified to maintain the comprehensive nature of the TDF. This could be done by selecting domains’ related key constructs as provided by Michie *et al.*[31] and selecting items from existing validated scales.

Lastly, the findings indicate that further refinement of the final questionnaire is required. In general, the amount of items measuring most of the domains could be increased to at least three items for each domain (at least three items with a loading above .80 will give a reliable component [64]). With regard to the specific domains, the final items measuring the domain *Environmental context and resources* are framed entirely in terms of the socio-political context, while there may be additional environmental and resources influences that remain unmeasured. The initial version of the questionnaire included items related to characteristics of the innovation, organization, socio-political context, and innovation strategies [16-20], however, only the items assessing the socio-political context were judged to discriminately assess this domain. Lack of discriminant content validity of items measuring characteristics of the innovation, organization, and innovation strategies might be due to our method of developing a generic questionnaire based on factors related to a specific implementation behavior (*i.e.*, the implementation of PA interventions). Moreover, the domain *Environmental context and resources* is arguably among the least well conceptualized domains of the TDF, which may partly explain challenges that judges faced in allocating items to this domain. Nevertheless, potential users of the final questionnaire may wish to incorporate additional more contextually sensitive items focusing on the environment and resources whilst recognizing that their discriminant content validity has not yet been demonstrated. In the initial questionnaire, items measuring the domain *Emotion* were adapted from previously published questionnaires. Specifically, items measuring the construct Affect were based on the Positive and Negative Affect Schedule [49] and Stress items were based on the General Health Questionnaire [48]. Items measuring the construct Stress demonstrated to be able to discriminately assess the domain *Emotions*, while Affect items did not. Therefore, the final questionnaire includes items concerning healthcare professionals’ general feelings (*i.e.*, Stress) instead of their emotions related to performing a specific behavior (*i.e.*, Affect). Yet, when investigating determinants of healthcare professionals’ implementation behaviors, items assessing emotions in relation to performing a specific behavior should also be taken into account as these have been found to be linked to implementation behaviors in previous research [65-67]. Although initial TACT-specific items assessing the construct Affect were not judged to discriminately assess the domain *Emotions*, potential users of the final questionnaire may want to consider using such items by including other emotions such as pride, empathy [67], fear [65-67], and embarrassment [66]. Furthermore, the assessment of the domain *Knowledge* could be improved by adding items to *test* healthcare professionals’ knowledge on a certain implementation behavior [66,68].

### Strengths and limitations of assessing TDF domains using questionnaires

Limitations with regard to the use of the TDF for questionnaire development involve the large amount of domains and underlying constructs that can only be assessed by a large amount of items. Quantitative TDF-based research might preclude measuring all constructs within each domain due to time constraints as described earlier by Amemori *et al.*[34]. As a result, it is not clear which constructs to choose when measuring a given domain. In this study, constructs were selected based on close relatedness to the content of the domains, being a part of important theories of behavior change, existence of validated scales, and/or relevance to the implementation of PA interventions in routine healthcare as determined in previous studies [43,44]. However, it is unclear to what extent the constructs that we selected measure the full breadth of the domains instead of a part of them. This questionnaire strove to balance representation of the constructs within the domains with a parsimonious questionnaire that could be feasibly used in the field. However, some domains cover a wider breath of constructs than others and future work could investigate the broader range of constructs within each domain. In addition, the TDF domains are *potential* behavioral determinants, instead of factors proven to influence implementation behavior and the framework does not specify relationships between domains [30]. On the other hand, quantitative applications of the framework can be beneficial for use in exploratory research and to guide theory selection.

Corresponding with the major rationale for the development of the original TDF, the framework can be used to assess a broad range of factors from a multitude of behavior change theories, helpful when little *a priori* information is available to base the selection of appropriate theories on. In comparison with other frameworks used in implementation research, *e.g.*, [16,17,20], and empirical work on the introduction of PA interventions in primary healthcare [43,44] the TDF [37], however, mainly focuses on factors related to the adopting person, instead of taking into account a variety of factors related to characteristics of the innovation, patient, social setting, organizational context, and innovation methods and strategies [16-20]. This implies factors outside psychological behavior change theory are not adequately elaborated in the framework. We believe that these factors may be included in the domain *Environmental context and resources* or multiple 'environmental’ domains should be incorporated in the TDF.

### Strengths and limitations of our methods

While we used a rigorous DCV approach to validate the content of items in the questionnaire, some limitations of our study need to be taken into account. The DCV exercise of allocating 79 items to 14 domains was a challenging task for judges, requiring consideration of multiple possible definitions. This approach is a degree of magnitude more challenging than how DCVs have typically been applied in the past (to a much smaller number of constructs). A larger number of judges and a less complex task would have possibly increased information on discriminant content validity of the items. Major strengths of this study include the sample of academics with expertise on TDF domains and the formulation of items using the 'TACT principle’ [38], which allows potential users of the questionnaire to tailor the content to their own target, action, context, and time. However, the operationalization and validation of the domains of the TDF are limited to these specific methods. It could be, for example, that in 'real life’ the validity of the domains would differ from the one perceived by an academic audience. Therefore, this study represents an important *first step* in the thorough development of a questionnaire to measure TDF-based factors underlying healthcare professionals’ implementation behaviors. As a next step we tested the Determinants of Implementation Behavior Questionnaire (DIBQ) on a sample of 270 healthcare professionals with specification of a particular target, action, context, and time, and showed good construct validity, with the majority of domains showing high internal consistency reliability and discriminant validity (Huijg *et al.*, submitted).

## Conclusions

To our knowledge, this study is the first to develop a generic (*i.e.*, formulation of items following the 'TACT principle’ [38]) TDF-based questionnaire in both English and Dutch including items which are able to discriminately measure a majority of the domains. The results partly support Cane *et al.*’s validation of the TDF [37] and suggest that the 12-domain version [31] might be more applicable in developing a TDF-based questionnaire. The items of this questionnaire can be used for the development of a questionnaire to measure TDF-based determinants of healthcare professionals’ specific implementation behaviors. Future research should investigate the concurrent and predictive validity and reliability of such a questionnaire in practice, among a large healthcare professional sample.

In general, a valid TDF-based questionnaire will increase the use of theory in the assessment of barriers and facilitators for implementation problems [31,69,70], which can inform the selection of possible techniques that can be used to change healthcare professionals’ behaviors [15,23,26]. Consequently, research on the development of a generic TDF questionnaire will improve our understanding of factors influencing healthcare professionals’ implementation and advance theory and methods in implementation research.

## Abbreviations

PA: Physical activity; TDF: Theoretical domains framework; DCV: Discriminant content validation; DIBQ: Determinants of implementation behavior questionnaire

## Competing interests

The authors declare that they have no competing interests.

## Authors’ contributions

JP and JMH are equally responsible for the design of the study, development of the questionnaire, and data analysis and interpretation. JMH collected the data and wrote the initial draft of the manuscript. Subsequent drafts of the manuscript were written together. WAG and MRC were involved in the development of the questionnaire and critically revised the manuscript. ED was involved in data analysis and interpretation, and commented on the manuscript. All authors read and approved the final manuscript.

## Supplementary Material

Additional file 1 Questionnaire items and related constructs and domains. Click here for file
