# Supplementary material for: Discriminant content validity of a theoretical domains framework questionnaire for use in implementation research
Source: Implement Sci. 2014 Jan 15;9:11. doi: 10.1186/1748-5908-9-11 (PMC3896680; doi:10.1186/1748-5908-9-11)
Supplement: Additional file 1 — Questionnaire items and related constructs and domains. [file 1748-5908-9-11-S1.docx]

**Additional file**

**Additional file 1 – Questionnaire items and related constructs and domains**

| **Domain** | | **Construct** | **Item** | **Source** |
| --- | --- | --- | --- | --- |
| D1 | Knowledge | Knowledge (3) | I am aware of the content and objectives of [innovation/guideline] | Adapted from Amemori et al. [34] and Beenstock et al. [35] |
|  |  |  | I know the content and objectives of [innovation, guideline] |  |
|  |  |  | I am familiar with the content and objectives of [innovation/guideline] |  |
|  |  | Procedural knowledge (3) | I am aware of how to [A] in [C, T] with [Ta] |  |
|  |  |  | I know how to [A] in [C, T] with [Ta] |  |
|  |  |  | I am familiar with how to [A] in [C, T] with [Ta] |  |
|  |  |  | (Strongly disagree – strongly agree) |  |
| D2 | Skills | Skills (4) | I have been trained how to [A] in [C, T] with [Ta] | New items |
|  |  |  | I have the proficiency to [A] in [C, T] with [Ta] |  |
|  |  |  | I have the skills to [A] in [C, T] with [Ta] |  |
|  |  |  | I have practiced [A] in [C, T] with [Ta] |  |
|  |  |  | (Strongly disagree – strongly agree) |  |
| D3 | Social/ professional role and identity | Professional role (4) | [A] in [C, T] with [Ta] is part of my work as a [profession] | New items |
|  |  |  | As a [profession], it is my job to [A] in [C, T] with [Ta] |  |
|  |  |  | It is my responsibility as a [profession] to [A] in [C, T] with [Ta] |  |
|  |  |  | Doing [A] in [C, T] with [Ta] is consistent with my [profession] |  |
|  |  |  | (Strongly disagree – strongly agree) |  |
| D4 | Beliefs about capabilities | Self-efficacy (2) | I am confident that I can [A] in [C, T] with [Ta] even when [Ta] is not motivated | Adapted from Bandura [42] |
|  |  |  | I am confident that I can [A] in [C, T] with [Ta] even when there is little time |  |
|  |  | Perceived behavioral control (4) | I am confident that if I wanted I could [A] in [C, T] with [Ta] | Adapted from Ajzen [41] |
|  |  |  | (Strongly disagree – strongly agree) |  |
|  |  |  | How much control do you have over [A] in [C, T] with [Ta]? (No control at all – a lot of control) |  |
|  |  |  | For me, [A] in [C, T] with [Ta] is… (Very difficult – very easy) |  |
|  |  |  | For me, [A] in [C, T] with [Ta] is… (Impossible – possible) |  |
| D5 | Optimism | Optimism (3) | With regard to [A] in [C, T] with [Ta] in uncertain Times, I usually expect the best | Adapted from Scheier et al. [52] |
|  |  |  | With regard to [A] in [C, T] with [Ta] I’m always optimistic about the future |  |
|  |  |  | With regard to [A] in [C, T] with [Ta] overall, I expect more good things to happen than bad |  |
|  |  | Pessimism (3) | With regard to [A] in [C, T] with [Ta] if something can go wrong, it will |  |
|  |  |  | With regard to [A] in [C, T] with [Ta] I hardly ever expect things to go my way |  |
|  |  |  | With regard to [A] in [C, T] with [Ta] I rarely count on good things happening to me |  |
|  |  |  | (Strongly disagree – strongly agree) |  |
| D6 | Beliefs about consequences | Attitudes (2) | For me, [A] in [C, T] with [Ta] is… (Useless – useful) | Adapted from Ajzen [41] |
|  |  |  | For me, [A] in [C, T] with [Ta] is… (Bad – good) |  |
|  |  | Outcome expectancies (2) | If I [A] in [C, T] with [Ta] it will benefit public health | Adapted from Bandura [42] |
|  |  |  | If I [A] in [C, T] with [Ta] it will have disadvantages for my relationship with [Ta] |  |
|  |  |  | (Strongly disagree – strongly agree) |  |
| D7 | Reinforcement | Reinforcement (3) | Whenever I [A] in [C, T] with [Ta], I get financial reimbursement | New items |
|  |  |  | Whenever I [A] in [C, T] with [Ta], I get recognition from professionals who are important to me |  |
|  |  |  | If I [A] in [C, T] with [Ta], I feel like I am making a difference |  |
|  |  |  | (Never – always) |  |
| D8 | Intentions | Intention (4) | For how many of the next 10 [Ta] do you intend to [A] in [C]? (1 – 10) | Adapted from Ajzen [41] |
|  |  |  | I will definitely [A] in [C] with [Ta]in the next [T] |  |
|  |  |  | I intend to [A] in [C] with [Ta] in the next [T] |  |
|  |  |  | (Strongly disagree – strongly agree) |  |
|  |  |  | How strong is your intention to [A] with [Ta] in [C] in the next [T]? (Not strong at all – very strong) |  |
| D9 | Goals | Action planning (4) | I have a clear plan of how I will [A] in [C, T] with [Ta] | Adapted from Sniehotta et al. [46] |
|  |  |  | I have a clear plan under what circumstances I will [A] in [C, T] with [Ta] |  |
|  |  |  | I have a clear plan when I will [A] in [C, T] with [Ta] |  |
|  |  |  | I have a clear plan how often I will [A] in [C, T] with [Ta] |  |
|  |  |  | (Strongly disagree – strongly agree) |  |
|  |  | Priority (4) | Generally, in [C, T] with [Ta], how often is covering something else on your agenda a higher priority than [A] | New items |
|  |  |  | Generally, in [C, T] with [Ta], how often does covering something else on your agenda take precedence over [A] |  |
|  |  |  | Generally, in [C, T] with [Ta], how often is covering something else on your agenda more urgent than [A] |  |
|  |  |  | Generally, in [C, T] with [Ta], how often is covering something else on your agenda more pressing than [A] |  |
|  |  |  | (Never – always) |  |
| D10 | Memory, attention and decision processes | Memory (4) | [A] in [C, T] with [Ta] is easy to remember | New items |
|  |  |  | (Strongly disagree – strongly agree) |  |
|  |  |  | How often do you forget [A] in [C, T] with [Ta]? |  |
|  |  |  | How often do you have to check the [innovation/guideline] before [A] in [C, T] with [Ta]? |  |
|  |  |  | (Never – almost always) |  |
|  |  |  | To what extent do you know [innovation/guideline] by heart to [A] in [C, T] with [Ta]? |  |
|  |  |  | (Not at all – very much so) |  |
|  |  | Attention (4) | When I need to concentrate to [A] in [C, T] with [Ta], I have no trouble focusing my attention | Adapted from Derryberry and Reed [51] |
|  |  |  | When I am working hard on [A] in [C, T] with [Ta], I still get distracted by events around me |  |
|  |  |  | When trying to focus my attention on [A] in [C, T] with [Ta], I have difficulty blocking out distracting thoughts |  |
|  |  |  | When concentrating on [A] in [C, T] with [Ta], I can focus my attention so that I become unaware of what’s going on around me |  |
|  |  |  | (Strongly disagree – strongly agree) |  |
| D11 | Environmental context and resources | Resources/ material (8) | [Innovation/guideline] has a good fit with routine practice | New items |
|  |  |  | [Innovation/guideline] provides the possibility to adapt it to the [Ta]’s needs (e.g., culture) |  |
|  |  |  | In the organization I work [A] in [C, T] with [Ta] is routine |  |
|  |  |  | In the organization I work there is enough time to [A] in [C, T] with [Ta] |  |
|  |  |  | Within the socio-political context there is sufficient financial support (e.g., from local authorities, insurance companies, the government) for [innovation/guideline] |  |
|  |  |  | Within the socio-political context there are good networks between parties involved in [innovation/guideline] |  |
|  |  |  | Prior to delivery of [innovation/guideline] professionals are provided with a training to [A] in [C, T] with [Ta] |  |
|  |  |  | During the delivery of [innovation/guideline] professionals are provided with sufficient financial reimbursement to [A] in [C, T] with [Ta] |  |
|  |  |  | (Strongly disagree – strongly agree) |  |
| D12 | Social influences | Social support (4) | I can rely on the team of professionals with whom I deliver [innovation] when things get tough on [A] in [C, T] with [Ta] | Adapted from Frese [50] |
|  |  |  | My colleagues are willing to listen to my problems related to [A] in [C, T] with [Ta] |  |
|  |  |  | The team of professionals with whom I deliver [innovation] is helpful in getting [A] in [C, T] with [Ta] done |  |
|  |  |  | I can rely on my colleagues when things get tough on [A] in [C, T] with [Ta] |  |
|  |  | Subjective norm (2) | Most people who are important to me think that I should [A] in [C, T] with [Ta] | Adapted from Ajzen [41] |
|  |  |  | Most people whose opinion I value would approve me of [A] in [C, T] with [Ta] |  |
|  |  | Descriptive norm (2) | The team of professionals with whom I deliver [innovation/guideline] [A] in [C, T] with [Ta] | Adapted from Cialdini et al. [53] |
|  |  |  | Respected colleagues [A] in [C, T] with [Ta] |  |
|  |  |  | (Strongly disagree – strongly agree) |  |
| D13 | Emotion | Affect (2) | Thinking about yourself and how you normally feel as a professional that delivers [innovation/guideline], to what extent do you generally feel inspired with regard to [A] in [C, T] with [Ta] | Adapted from Thompson [49] |
|  |  |  | Thinking about yourself and how you normally feel as a professional that delivers [innovation/guideline], to what extent do you generally feel nervous with regard to [A] in [C, T] with [Ta] |  |
|  |  | Stress (2) | Have you recently, during the past two weeks been able to enjoy your normal day-to-day activities? | Goldberg and Blackwell [48] |
|  |  |  | Have you recently, during the past two weeks been feeling unhappy and depressed? |  |
|  |  |  | (Never always) |  |
| D14 | Behavioral regulation | Automaticity (2) | [A] in [C, T] with [Ta] is something I do automatically | Adapted from Gardner et al. [47] |
|  |  |  | [A] in [C, T] with [Ta] is something I do without thinking |  |
|  |  | Self-monitoring (4) | I keep track of my overall progress towards [A] in [C, T] with [Ta] | Adapted from Maes et al. [45] |
|  |  |  | I tend to notice my successes while working towards [A] in [C, T] with [Ta] |  |
|  |  |  | I am aware of my day-to-day behavior as I work towards [A] in [C, T] with [Ta] |  |
|  |  |  | I check regularly whether I am getting closer to attaining [A] in [C, T] with [Ta] |  |
|  |  | Action planning (4) | I have a clear plan of how I will [A] in [C, T] with [Ta] | Adapted from Sniehotta et al. [46] |
|  |  |  | I have a clear plan under what circumstances I will [A] in [C, T] with [Ta] |  |
|  |  |  | I have a clear plan when I will [A] in [C, T] with [Ta] |  |
|  |  |  | I have a clear plan how often I will [A] in [C, T] with [Ta] |  |
|  |  |  | (Strongly disagree – strongly agree) |  |
| *Note.* [A], action; [C], context; [T], time; [Ta], target | | | | |
